# Medical Corps U. S. Army

**Published:** 1883-05

**Authors:** 


### Medical Corps U. S. Army.

An Army Medical Board has been ordered to assemble at the Army Building corner of Houston and Greene Streets, New York City, New York, March 1, 1883, for the examination of such persons as may be properly invited to present themselves before it as candidates for appointment in the Medical Corps of the Army, and will probably continue in session about three months.

All candidates for appointment in the Medical Corps must apply to the Secretary of War for an invitation to appear for examination. The application must be in the handwriting of the applicant, must state date and place of his birth and place and state of which he is a permanent resident, and must be accompanied by certificates based on personal acquaintance from at least two persons of repute as to citizenship, character and moral habits; testimonials as to professional standing from Professors of the medical college at which they graduated, should also accompany the application if they can be obtained. The candidate must be between 21 and 28 years of age (without any exceptions) and a graduate of a regular medical college, evidence of which, his Diploma, must be submitted to the Board.

Further information regarding these examinations and the nature thereof, can be obtained by addressing the Surgeon General, U. S. Army, Washington, D. C.

